# Preload dependence indices to titrate volume expansion during septic shock: a randomized controlled trial

**DOI:** 10.1186/s13054-014-0734-3

**Published:** 2015-01-08

**Authors:** Jean-Christophe Richard, Frédérique Bayle, Gael Bourdin, Véronique Leray, Sophie Debord, Bertrand Delannoy, Alina Cividjian Stoian, Florent Wallet, Hodane Yonis, Claude Guerin

**Affiliations:** Service de Réanimation Médicale, Hôpital De La Croix Rousse, Hospices Civils de Lyon, 103 Grande Rue de la Croix Rousse, 69004 Lyon, France; Université de Lyon, Université LYON I, 37 Rue du Repos, 69007 Lyon, France; CREATIS INSERM 1044 CNRS 5220, 20 Avenue Albert Einstein, 69621 Villeurbanne, France

## Abstract

**Introduction:**

In septic shock, pulse pressure or cardiac output variation during passive leg raising are preload dependence indices reliable at predicting fluid responsiveness. Therefore, they may help to identify those patients who need intravascular volume expansion, while avoiding unnecessary fluid administration in the other patients. However, whether their use improves septic shock prognosis remains unknown. The aim of this study was to assess the clinical benefits of using preload dependence indices to titrate intravascular fluids during septic shock.

**Methods:**

In a single-center randomized controlled trial, 60 septic shock patients were allocated to preload dependence indices-guided (preload dependence group) or central venous pressure-guided (control group) intravascular volume expansion with 30 patients in each group. The primary end point was time to shock resolution, defined by vasopressor weaning.

**Results:**

There was no significant difference in time to shock resolution between groups (median (interquartile range) 2.0 (1.2 to 3.1) versus 2.3 (1.4 to 5.6) days in control and preload dependence groups, respectively). The daily amount of fluids administered for intravascular volume expansion was higher in the control than in the preload dependence group (917 (639 to 1,511) versus 383 (211 to 604) mL, *P* = 0.01), and the same held true for red cell transfusions (178 (82 to 304) versus 103 (0 to 183) mL, *P* = 0.04). Physiologic variable values did not change over time between groups, except for plasma lactate (time over group interaction, *P* <0.01). Mortality was not significantly different between groups (23% in the preload dependence group versus 47% in the control group, *P* = 0.10). Intravascular volume expansion was lower in the preload dependence group for patients with lower simplified acute physiology score II (SAPS II), and the opposite was found for patients in the upper two SAPS II quartiles. The amount of intravascular volume expansion did not change across the quartiles of severity in the control group, but steadily increased with severity in the preload dependence group.

**Conclusions:**

In patients with septic shock, titrating intravascular volume expansion with preload dependence indices did not change time to shock resolution, but resulted in less daily fluids intake, including red blood cells, without worsening patient outcome.

**Trial registration:**

Clinicaltrials.gov NCT01972828. Registered 11 October 2013.

**Electronic supplementary material:**

The online version of this article (doi:10.1186/s13054-014-0734-3) contains supplementary material, which is available to authorized users.

## Introduction

Fluid administration is the first-line component of hemodynamic support in septic shock treatment [1]. Preload optimization is a major issue in the treatment of these patients. However, observational studies found a strong association between intensive care unit (ICU) mortality and positive fluid balance [2,3], suggesting that aggressive fluid resuscitation may be harmful. While several hemodynamic algorithms have been evaluated in randomized controlled trials during the first hours of septic shock resuscitation [4-7], the evidence is relatively scarce regarding the practical modalities of fluid administration some hours later. A substantial amount of physiological studies [8,9] have demonstrated that static preload indices (such as central venous pressure (CVP)) may not be reliable to assess fluid responsiveness (cardiac output change in response to fluid administration), especially in septic patients. In contrast, dynamic preload indices such as pulse pressure variation (PPV) or stroke volume change during passive leg raising (PLR) are highly reliable to assess fluid responsiveness [10,11], should validity conditions for PPV accuracy be met. Driving intravascular volume expansion with dynamic preload indices should avoid unnecessary fluid administration, preventing pulmonary side effects, and select fluid-responsive patients. As a result, oxygen delivery should be optimized and organ failure shortened. However, while several studies have demonstrated a beneficial effect of volume expansion driven by preload dependence indices in the perioperative context [12-15], none has been performed in septic shock patients.

We conducted an exploratory randomized controlled study to explore whether preload dependence-driven fluid management in ICU patients with septic shock would reduce the duration of cardiovascular failure, as compared to CVP-driven fluid management.

## Materials and methods

### Study design

This trial was an open-label, controlled, parallel-group study, with balanced randomization, performed in a 15-bed medical ICU. The trial was registered at ClinicalTrials.gov under the number NCT01972828 and the study protocol was approved by the local ethics committee (Comité de Protection des Personnes Sud-Est III). Patients were enrolled between 1 July 2007 and 30 July 2013. The attending physician was responsible for enrolling the patients in the study, and following the protocol. Written informed consent was obtained from the patients themselves or their closest relatives, prior to their inclusion.

### Patients

Eligible participants were adults aged 18 years or older with septic shock [16], who had received intravascular fluid loading of at least 25 mL.kg^-1^ body weight, with hypotension onset less than 6 hours before inclusion. An amendment to the original protocol extended the time from hypotension onset to 12 hours after inclusion of the first three patients.

Exclusion criteria were pregnancy, acute coronary syndrome, acute cerebral event during the previous 30 days, cardiogenic pulmonary edema, contraindication to central venous or femoral artery catheterization, uncontrolled hemorrhage, burn injury on more than 20% of the body surface, trauma, requirement for immediate surgery or radiological procedure, previous inclusion in present study, inclusion in another randomized controlled trial during the same ICU stay, advance directives to withhold or withdraw life-sustaining treatment, lack of written informed consent by patient or next of kin, lack of affiliation to social security as required by French regulation, patient under a legal protective measure.

### Randomization

Patients were randomized into a control group, and a preload dependence group, with a computer-generated list using a 1:1 ratio. The allocation sequence was generated and concealed from the enrolling physician in sequentially numbered, opaque, and sealed envelopes, by a co-author (CG) who did not participate in the assessment of patient eligibility for the study.

### Protocol description

**Figure 1:**
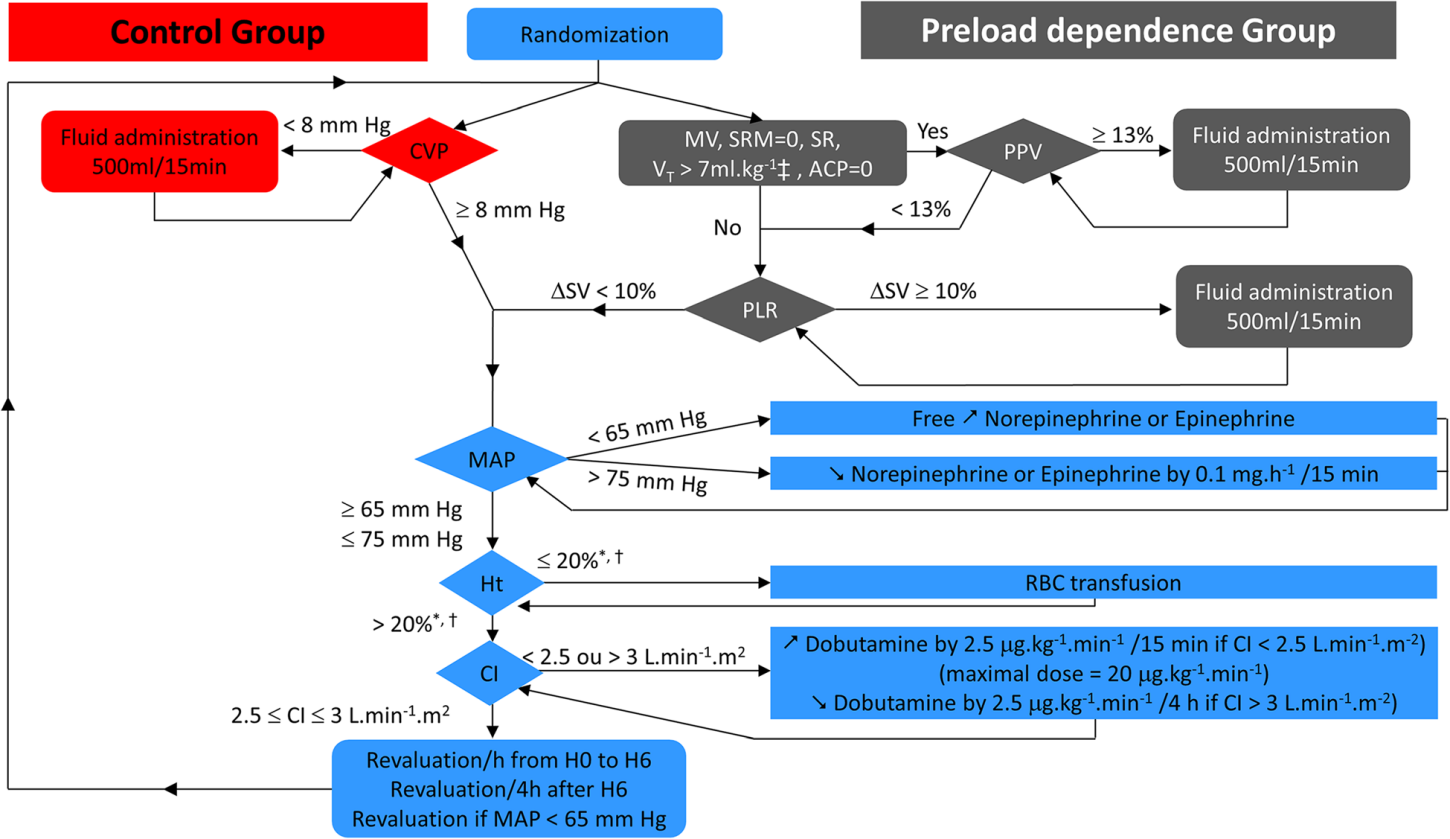
**Treatment algorithm.** ^*^or hemoglobin <7 g.dL^-1^; †Ht ≤30% or hemoglobin ≤10 g.dL^-1^ in the first 6 hours following inclusion; ^‡^ml.kg^-1^ of predicted body weight. ACP, acute cor pulmonale; CI, cardiac index; CVP, central venous pressure; Ht, hematocrit; MAP, mean arterial pressure; MV, mechanical ventilation; PLR, passive leg raising test; PPV, pulse pressure variation; RBC, red blood cells; SR, sinus rhythm; SRM, spontaneous respiratory movements; ΔSV, stroke volume variation after fluid administration; V_T_, tidal volume.

Jugular central venous and femoral arterial lines were connected to the Picco plus device (Pulsion Medical Systems, Munich, Germany) for the first 31 patients, or an Intellivue MP40 monitor equipped with the Picco-Technology module thereafter (Philips Healthcare, Andover, MA, USA). Calibration of cardiac output was performed by intravenous infusion of 15 mL serum saline in triplicate, every hour during the first 6 hours of the study, and every 4 hours thereafter. Hemodynamic treatments were administered according to an hemodynamic algorithm (Figure 1), run at each episode of hypotension (defined by mean arterial pressure (MAP) below 65 mm Hg), every hour during the first 6 hours after inclusion, and every 4 hours thereafter until vasopressor weaning (defined by the absence of vasopressor reintroduction for at least 24 hours). Intravascular volume expansion was performed using CVP in the control group, and using both PPV when applicable (invasive mechanical ventilation (MV) with no spontaneous respiratory movement (SRM), sinus rhythm (SR), tidal volume (V_T_) greater than 7 ml.kg^-1^ of predicted body weight (PBW), and absence of evidence for acute cor pulmonale (ACP)), or stroke volume change (ΔSV) during PLR in the preload dependence group. PLR was performed from the supine position, for 1 minute, and preload dependence was deemed present if stroke volume increased by at least 10% during the procedure. Intravascular volume expansion was administered by aliquots of 500 ml over 15 minutes, to achieve a CVP of at least 8 cm H_2_O in the control group, and a PPV below 13% or a stroke volume increase below 10% during PLR in the preload dependence group. The type of fluid used for intravascular volume expansion was left at the discretion of the attending physician. In both groups, MAP was maintained between 65 and 75 mm Hg using standardized vasopressors management. The same hematocrit (Ht) and cardiac index (CI) targets were used in both groups.

Concomitant treatments were administered according to international guidelines at the time of study design [17] (antibiotherapy within the first hour of recognition of septic shock, source control, low-dose steroids for 7 days, MV in volume assist-control mode with V_T_ 6 to 8 ml.kg^-1^ PBW and plateau pressure below or equal to 30 cm H_2_O, positive end-expiratory pressure (PEEP) and inspired oxygen fraction (FiO_2_) titrated using a PEEP-FIO_2_ table to achieve 88 to 95% pulse oximetry or 55 to 80 mm Hg partial pressure of arterial oxygen (PaO_2_) [18]). Sedation was reevaluated every day by the attending physician. Weaning from MV was performed daily by attending nurses, according to local protocol. Glucose control was performed by intravenous (IV) insulin targeting glycemia in the range 6 to 8.3 mmol.L^-1^. Diuretics use or fluid removal by hemodialysis was performed in case of fluid overload, and not recommended in the first 48 hours of septic shock.

### Data collection and follow-up

The following variables were recorded at inclusion: demographic and anthropometric data, time of hospital and ICU admission, context for admission to the ICU, immunodeficiency, Charlson comorbidity score [19], vital signs, Simplified Acute Physiology Score (SAPS) II [20], delay from hypotension onset and inclusion, volume of fluids administered for intravascular volume expansion between hypotension onset and inclusion.

The following variables were recorded at time of inclusion, 6 and 12 hours later and daily during follow-up: Sequential Organ Failure Assessment (SOFA) score [21], vital signs, CVP, CI, extravascular lung water index, arterial lactate, arterial and venous blood gases, standard laboratory tests, need for MV or renal replacement therapy, dose of vasopressor (norepinephrine plus epinephrine) or dobutamine, volume and type of fluid used for volume expansion, red blood cell (RBC) transfusion requirement, fluid balance, PEEP level, FiO_2_ and V_T_ if applicable, and other relevant therapeutic interventions.

The following variables were recorded at the time of availability: source of infection, results of microbiological culture, adequacy of initial empirical antibiotherapy (defined as an initial antimicrobial regimen with *in vitro* activity against one or more pathogens that were judged to be responsible for the infection), and time to successful extubation (defined as no reintubation within the 48 hours following extubation).

Hemodynamic data were reported by nurses at each assessment of the hemodynamic algorithm.

Patients were followed up until occurrence of one of the following events: vasopressor weaning and lactate normalization for at least 24 hours, 28 days after inclusion, or in case of patient death.

### Study outcomes

The primary outcome was time to shock resolution, defined by vasopressor weaning. Secondary outcomes were the followings: ventilator-free days at day 28, number of days with greater than normal plasma lactate, pulmonary edema (that is extravascular lung water index (ELWI) >10 ml.kg^-1^ PBW) or organ system failure (that is SOFA ≥6) from inclusion to end of study, ICU length of stay, and mortality at day 28.

### Assessment of data quality

All individual data were independently checked for accuracy by the Delegation à la Recherche Clinique des Hospices Civils de Lyon. Quality audits included validity control of the informed consent, compliance to the protocol, validity of data recorded in the case report form compared with the medical charts, validity of outcome assessment and accuracy of serious adverse events reporting. One of the co-authors (JCR) checked the adherence to hemodynamic and associated treatments protocols on a daily basis, and reported protocol violation if any.

### Statistical analysis

The expected duration of shock in the control group was 9 ± 2 days [22]. We calculated that with a sample of 60 patients, the study would have an 80% power to detect an absolute reduction in shock duration of 1.5 days, using a two-sided test with a 0.05 type I error. The analysis was performed on an intention-to-treat basis, unless specifically stated. No imputation was performed for missing data. Median and interquartile range were reported for continuous variables, and number of patients in each category and corresponding percentages are given for categorical variables. Data were compared between groups with the chi-square or Fisher’s exact test for categorical variables and *t* test, ANOVA or Mann-Whitney *U* test for continuous or ordinal variables if indicated. The bias corrected and accelerated bootstrap method was used to compute confidence intervals for the difference in median times between groups [23]. Repeated physiological measurements over time were compared between groups with a linear mixed model using time as a continuous variable, group and their interaction as variables with fixed effects, and patient as variable with a random effect. The probability for remaining under vasopressor or to survive was analyzed with the Kaplan-Meier method, and compared between groups with the log-rank test. Multivariate analysis was performed using multiple linear regression and a backward selection algorithm. Statistical analyses were performed using R software [24], with packages nlme [25], simpleboot [26], survival [27] and prodlim [28].

## Results

**Figure 2:**
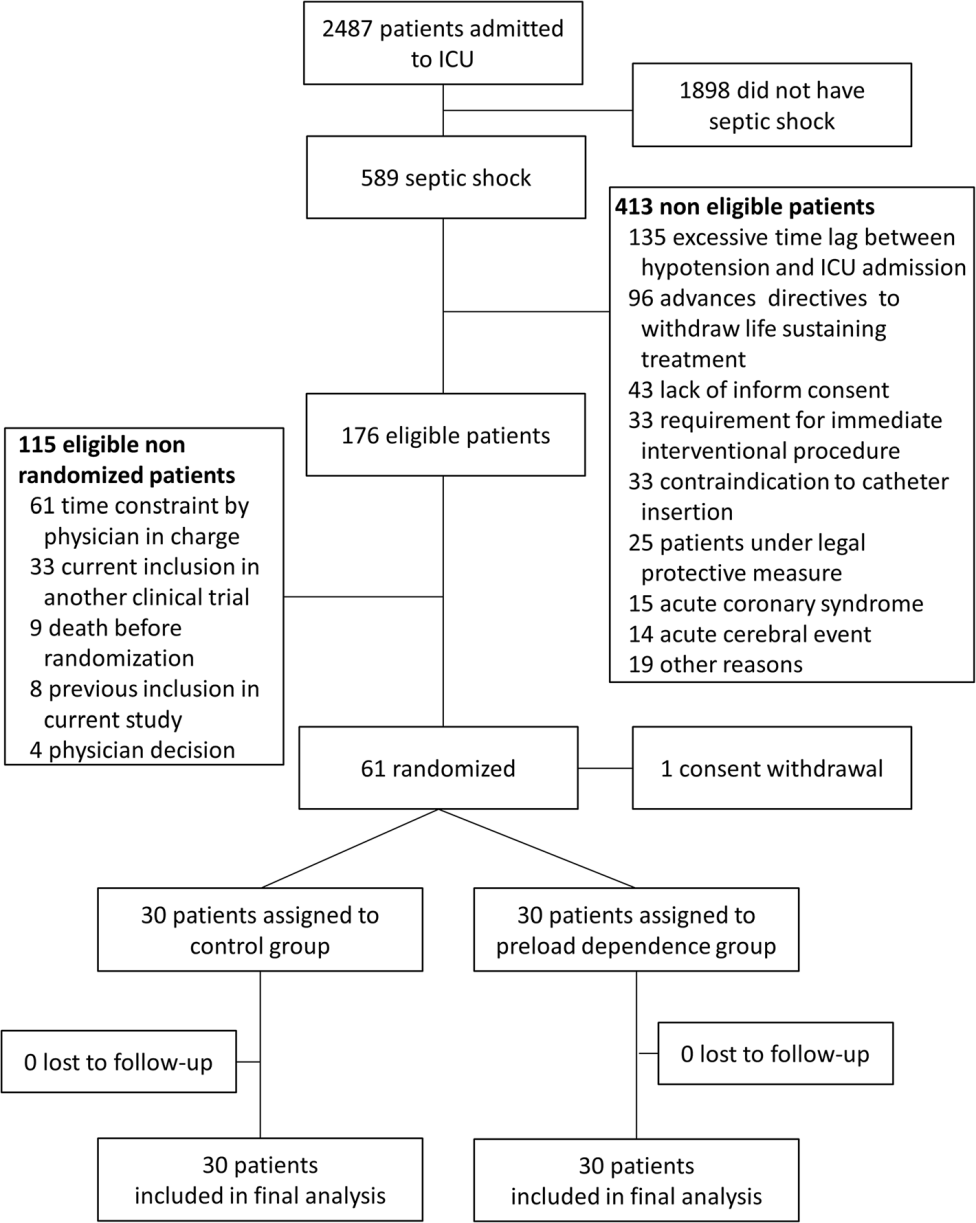
**Study flow chart.** ICU, intensive care unit.

During the study inclusion period, 589 patients were admitted with septic shock, and 61 randomized (Figure 2). One patient subsequently withdrew consent, leaving 60 patients for final analysis. No patient was lost to follow-up.

### Characteristics at inclusion

Both treatment arms were well balanced at both admission and inclusion (Tables 1 and 2). The total duration of the study (that is application of the hemodynamic algorithm) was 3.4 [2.6 to 4.5] and 3.4 [2.7 to 6.9] days in the control and preload dependence groups, respectively (*P* = 0.40).

**Table 1:** **General characteristics at admission and inclusion**

	**Control**	**Preload dependence**	***P***
	**(n = 30)**	**(n = 30)**	
Age (years)	64 [54-76]	65 [58-80]	0.38
Male gender	22 (73%)	21 (70%)	1
Admission category			
Medical	27 (90%)	30 (100%)	0.24
Unscheduled surgery	3 (10%)	0 (0%)	
Charlson score	3 [2-5]	3 [1-5]	0.74
Immunodeficiency^*^	9 (30%)	9 (30%)	1
SAPS II	56 [50-60]	57 [47-69]	0.55
SOFA score at inclusion	10 [9-12]	11 [9-13]	0.52
Time between hypotension and inclusion (H)	9 [5-11]	10 [6-11]	0.64
Time between ICU admission and inclusion (H)	8 [4-15]	6 [3-12]	0.55
Renal replacement therapy	6 (20%)	8 (27%)	0.76
Mechanical ventilation	26 (87%)	20 (67%)	0.13
Volume of IV fluids administered between hypotension and inclusion (L)	3.0 [2.5-4.0]	3.5 [2.5-4.4]	0.26
Type of infection			
Community-acquired infection	23 (77%)	21 (70%)	0.77
Hospital-acquired infection	7 (23%)	9 (30%)	
Infection site			
Pulmonary	16 (53%)	18 (60%)	0.80
Intra-abdominal	5 (17%)	7 (23%)	0.75
Urinary tract	3 (10%)	6 (20%)	0.47
Catheter-related infection	1 (3%)	3 (10%)	0.61
Other	3 (10%)	4 (13%)	1
Positive blood cultures	15 (50%)	15 (50%)	1
Identification of causative pathogen	25 (83%)	27 (90%)	0.71
Causative pathogens			
Enterobacteriaceae	13 (43%)	16 (53%)	0.61
Non-fermenting gram-negative bacilli	5 (17%)	3 (10%)	0.71
Other gram-negative bacilli	2 (7%)	4 (13%)	0.67
Staphylococci	6 (20%)	7 (23%)	1
Streptococci	2 (7%)	6 (20%)	0.25
Gram-positive bacilli	0 (0%)	1 (3%)	1
Enterococci	0 (0%)	1 (3%)	1
Gram-negative cocci	0 (0%)	1 (3%)	1
Fungi	0 (0%)	1 (3%)	1
Empirical antibiotic therapy			
Adequate	24 (80%)	23 (77%)	0.42
Inadequate	1 (3%)	4 (13%)	
Not applicable	5 (17%)	3 (10%)	

Data are median [interquartile range] or number of patients (%). ^*^immunodeficiency was considered in any of the following situations: chronic treatment with steroids or other immunosuppressive agents, chemotherapy within one month, infection with the human immunodeficiency virus, neutropenia below 0.5 G.L^-1^, or past history of splenectomy. SAPS II, Simplified Acute Physiology Score II [20]; SOFA, Sequential Organ Failure Assessment score [21]; ICU, intensive care unit; IV, intravenous.

**Table 2:** **Hemodynamic characteristics at inclusion**

	**Control**	**Preload dependence**	***P***
	**(n = 30)**	**(n = 30)**	
Mean arterial pressure (mm Hg)	68 [64-75]	72 [68-82]	0.08
Central venous pressure (mm Hg)	9 [7-14]	10 [8-12]	0.67
Cardiac index (L.min^-1^.m^-2^)	3.5 [2.7-4.7]	3.6 [2.9-4.8]	0.43
Hemoglobin (g.dL^-1^)	9.8 [8.9-11.6]	10.6 [9.5-11.4]	0.57
Oxygen arterial transport (mL.min^-1^.m^-2^)	414 [352-619]	468 [395-648]	0.14
ScvO_2_ (%)	77 [74-83]	77 [72-84]	0.89
ScvO_2_ < 70%	5 (17%)	6 (20%)	1
Extravascular lung water index (mL.kg^-1^ PBW)	13 [10-16]	12 [10-16]	0.65
Lactate (mmol.L^-1^)	2.7 [2.2-3.6]	2.9 [2.5-5.7]	0.32
Lactate above upper normal laboratory limit	22 (73%)	25 (83%)	0.53
Inotrope treatment	5 (17%)	6 (20%)	1
Vasopressor dose (μg.kg^-1^.min^-1^)	0.51 [0.26-1.05]	0.60 [0.34-1.14]	0.38

Data are median [interquartile range] or number of patients (%). ScvO_2_, superior vena cava oxygenation saturation; PBW, predicted body weight.

### Time course of hemodynamic parameters in both groups

**Figure 3:**
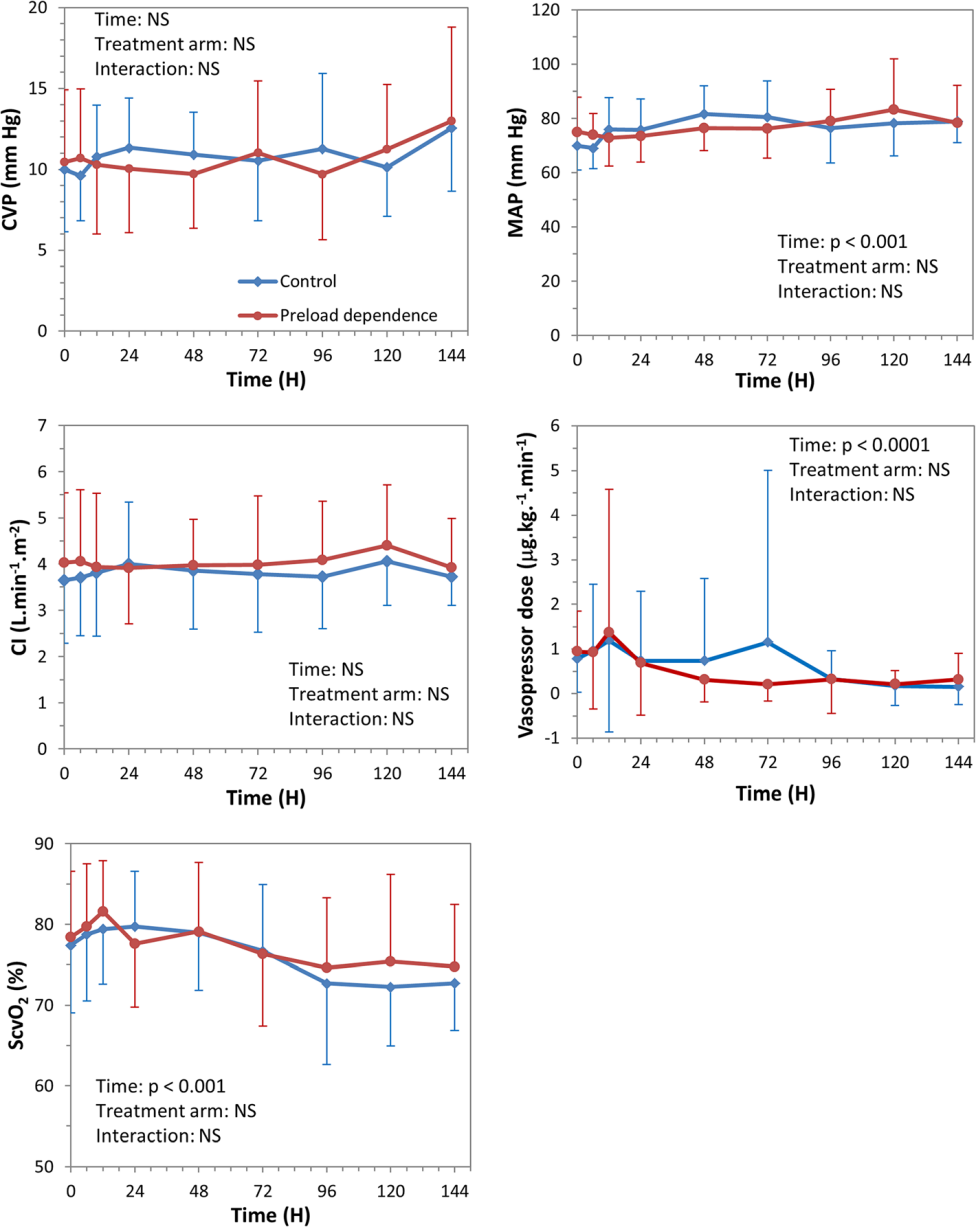
**Evolution of hemodynamic parameters over time.** Symbols are mean parameter values over time (blue = control group, red = preload dependence group). Bars are standard deviation. CI, cardiac index; CVP, central venous pressure; MAP, mean arterial pressure; NS, non-statistically significant; ScvO_2_, superior vena cava venous oxygen saturation.

**Figure 4:**
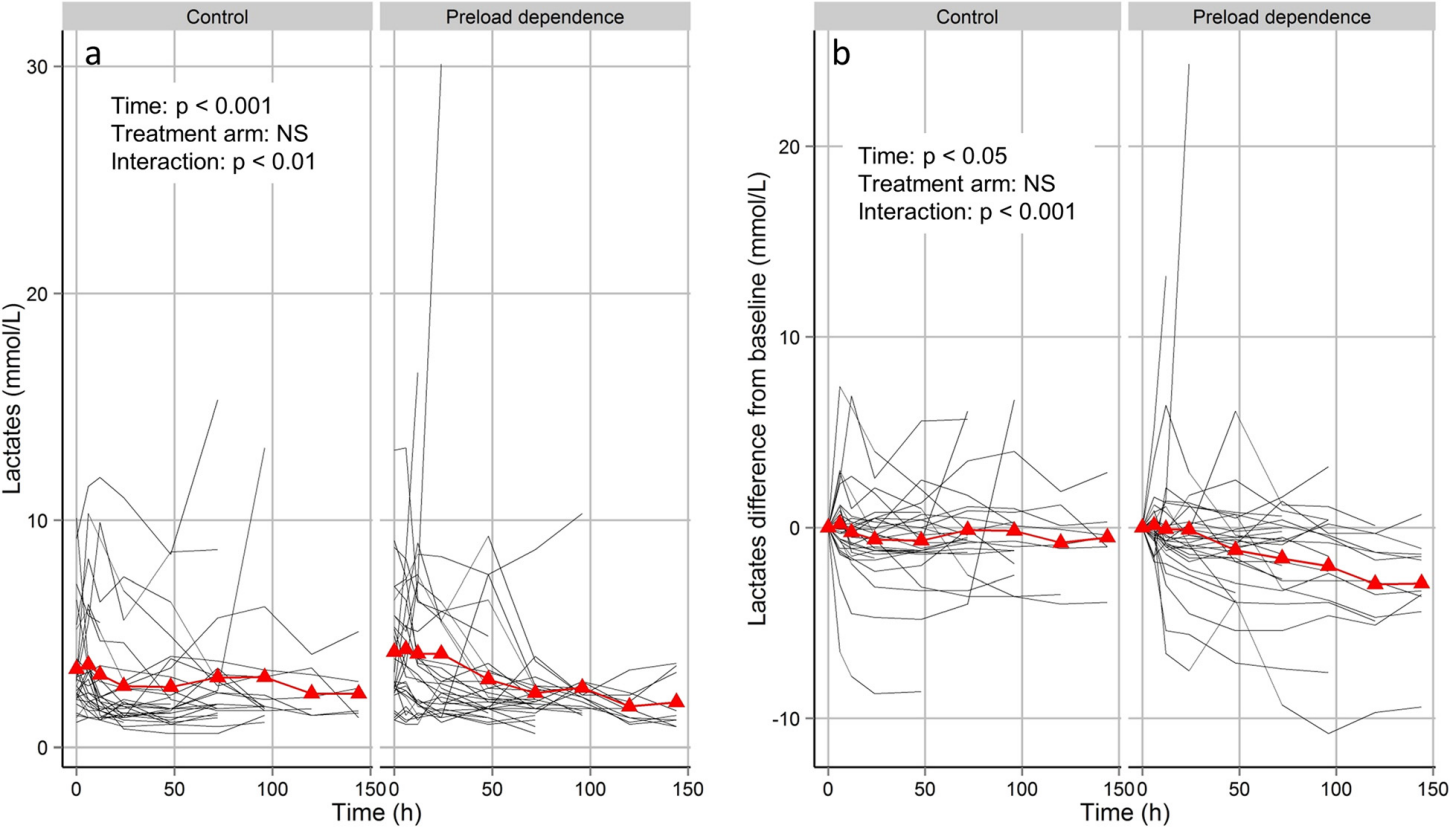
**Evolution of lactates over time (a) and lactate difference from inclusion (b) at each time point.** Red symbols are mean parameter values over time. Black lines are individual parameter values over time.

CVP and CI were not significantly different between groups, without significant variation over time (Figure 3). In both groups, MAP significantly increased over time, while superior vena cava oxygenation saturation (ScvO_2_) and vasopressor dose significantly decreased, without any significant difference between groups (Figure 3). A significant interaction between time and group was found for plasma lactate, indicating that the lactate decline over time was greater in the experimental group than in the control group (Figure 4a). Similar findings were observed for lactate difference from baseline (Figure 4b).

### Fluid administration and fluid balance in both groups

**Figure 5:**
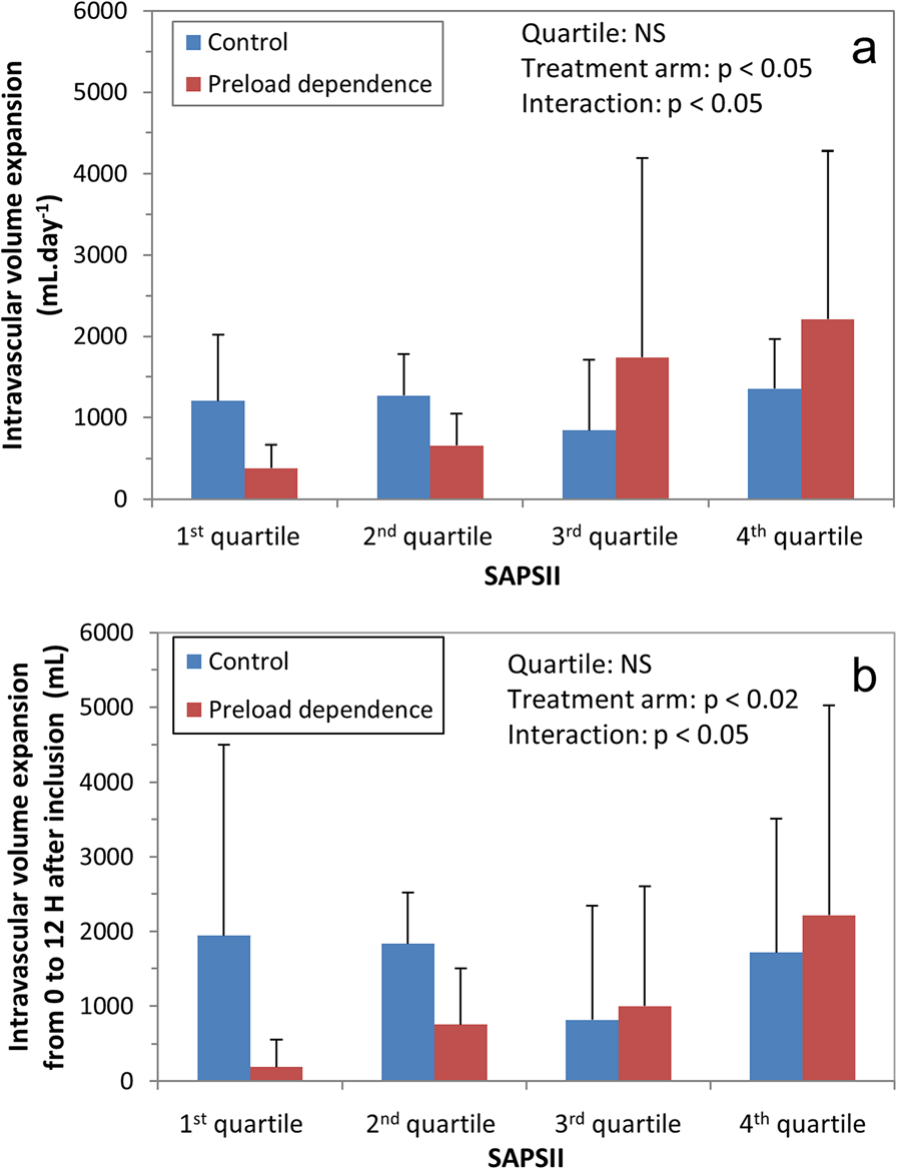
**Amount of fluids administered for intravascular volume expansion as a function of treatment arm and SAPS II. (a)** Daily amount of fluids. **(b)** Amount of fluid administered from H0 to H12 after inclusion. In both groups, patients were classified into four categories of severity at inclusion according to quartiles of SAPS II score [20]. Bars are mean values and error bars standard deviation. NS, non-statistically significant; SAPS II, Simplified Acute Physiology Score II.

In the preload dependence group, intravascular volume expansion was indicated from PPV and PLR criteria in 4% and 96% of the cases, respectively. The reason for this difference is that those criteria required for PPV use were present in only 9% of the total number of hemodynamic algorithm sessions. The daily amount of fluids administered per protocol for intravascular volume expansion was significantly higher in the control group (917 [639 to 1,511] vs. 383 [211 to 604] mL.day^-1^, *P* = 0.01, Table 3). Additional data regarding fluid administration as a function of time from inclusion are provided in Additional file 1. The daily amount of RBC transfusion was significantly lower in the preload dependence group (*P* = 0.04), while hemoglobin levels were not different between groups (see Additional file 2). There was no significant difference in intake of other fluids, total fluid intake, total fluid output and fluid balance (Table 3). Volume expansion was performed almost exclusively using crystalloids, with the exception of one patient in the control, and two in the preload dependence group who received at least 500 mL of hydroxylethyl starch during the study (*P* = 1). A multivariate analysis of variables associated with the daily amount of intravascular volume expansion was performed using treatment arm, SAPS II, and the following variables at time of inclusion as predictors: SOFA score, lactates, body weight). SAPS II, treatment arm and their interaction were the only significant variables retained in the final model. As shown on Figure 5a, intravascular volume expansion was lower in the intervention group for patients with lower SAPS II, and the opposite was found for patients pertaining to the upper two SAPS II quartiles. Furthermore, the amount of intravascular volume expansion was unchanged in the control group whatever the quartile of severity (lack of significance of the quartile effect), while a stepwise increase was observed as severity increased in the preload dependence group (as a result of the significant interaction). Similar results were observed using the amount of fluid administered for intravascular volume expansion during the first 12 hours of the study (Figure 5b). This time point was chosen as the last time point with all included patients.

**Table 3:** **Fluid administration and fluid balance**

	**Control**	**Preload dependence**	***P***
	**(n = 30)**	**(n = 30)**	
Intravascular volume expansion ITT (mL.day^-1^)	986 [654-1,624]	446 [295-1,105]	0.04
Intravascular volume expansion PP (mL.day^-1^)	917 [639-1,511]	383 [211-604]	0.01
RBC transfusion (mL.day^-1^)	178 [82-304]	103 (0-183]	0.04
Other blood products (mL.day^-1^)	0 [0-122]	0 [0-125]	0.76
Other fluids (mL.day^-1^)	3151 [2,791-3,456]	2919 [2,533-3,368]	0.70
Fluid intake^*^ (mL.day^-1^)	4096 [3,770-4,677]	3610 [2,982-4,560]	0.16
Diuresis (mL.day^-1^)	2116 [368-3,212]	1854 [513-3,332]	0.95
Fluid output (mL.day^-1^)	2550 [1,914-3,331]	2609 [2,079-3,202]	0.95
Fluid balance (mL.day^-1^)	1749 [146-2,788]	888 [153-2,816]	0.68
Intravascular volume expansion/fluid intake (%)	23%	15%	0.04

Data are median [interquartile range] or number of patients (%). ^*^total volume of fluids administered (intravascular volume expansion + blood products + other fluids). ITT, intention to treat; PP, per protocol; RBC, red blood cells.

### Associated treatments

Norepinephrine was used as a first-line vasopressor agent in all patients (see Additional file 3). Epinephrine was used as a second-line vasopressor agent for refractory shock, in four (13%) and three (10%) patients in the control and preload dependence groups, respectively (*P* = 1). Use of other associated treatments for septic shock was similar in both groups.

### Outcomes

There was no significant difference in time to shock resolution between groups (Table 4). The difference in median time to shock resolution between preload dependence and control groups was 0.3 days (95% confidence interval -0.8 to 2.1 days).

**Table 4:** **Study outcomes**

	**Control**	**Preload dependence**	***P***
	**(n = 30)**	**(n = 30)**	
Time to shock resolution (days)	2.0 [1.2-3.1]	2.3 [1.4-5.6]	0.29
Ventilator-free days at day 28	8 [0-21]	14 [0-24]	0.35
Number of days with lactates above upper normal laboratory limit	1 [1-4]	2 [1-4]	0.14
Number of days with pulmonary edema (that is ELWI >10 ml.kg^-1^ PBW)	4 [1-5]	4 [1-6]	0.94
Number of days with organ system failure (that is SOFA ≥6)	4 [3-5]	4 [2-8]	0.61
ICU length of stay (days)	10 [7-20]	14 [6-28]	0.55
In survivors	14 [9-28]	22 [6-28]	0.89
In non-survivors	8 [5-11]	5 [3-17]	0.85
Mortality at day 28	14 (47%)	7 (23%)	0.10

Data are median [interquartile range] or number of patients (%). ELWI, extravascular lung water index; ICU, intensive care unit; PBW, predicted body weight; SOFA, Sequential Organ Failure Assessment score [21].

**Figure 6:**
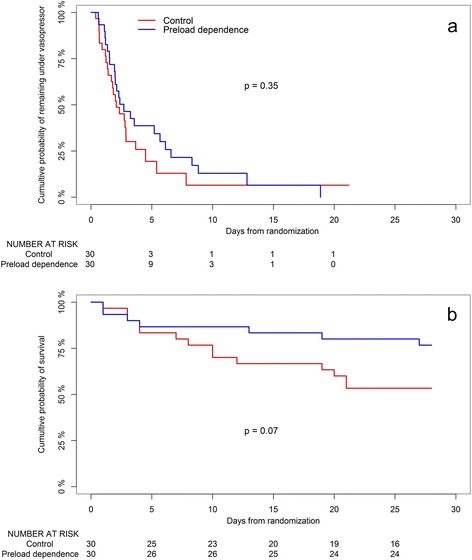
**Kaplan-Meier plot of the probability of remaining under vasopressor therapy (a) and survival (b) from inclusion to day 28.**

The probability of remaining under vasopressor until day 28 was not statistically different between groups (Figure 6a). Ventilator-free days tended to be higher in the preload dependence than in the control group (14 vs. 8, *P* = 0.35). Number of days with plasma lactate values higher than normal, pulmonary edema, or organ system failure and ICU length of stay were similar in both groups. Mortality was reduced by approximately 50% in the preload dependence group (23% vs. 47%), without reaching statistical significance (*P* = 0.10). Probability of survival until day 28 (Figure 6b) was not statistically significant (*P* = 0.07) between groups. There was no significant difference in cause of death or end-of-life decisions between groups (see Additional file 4).

### Protocol violations

There was no significant difference between treatment arms regarding protocol violations related to intravascular volume expansion (see Additional file 5). The median number of protocol violations per day amounted to 0 in both groups (*P* = 0.23). Nine (30%) patients in the control and 13 (43%) patients in the preload dependence group were exposed to more than one protocol violation between inclusion and end of study (*P* = 0.42).

## Discussion

This is the first randomized controlled study that investigates in medical ICU patients the effects of a strategy using preload dependence indices on both physiological end points and patient outcome. The main findings of this study are that titration of intravascular volume expansion by using preload dependence indices, as compared to CVP: (1) significantly reduces the daily amount of fluids intake and RBC transfusion, without adverse effect on outcome; (2) results in higher fluid administration in those patients with the most general severity score.

### Limitations and strengths

Before discussing present results, some limitations should be acknowledged. First, patients were enrolled relatively late in the course of septic shock. It should, however, be stressed that this may decrease the probability of detecting an effect of the intervention being tested in the present study, while we observed beneficial effects regarding fluid requirement and lactate decrease. Second, expected shock duration was overestimated when computation of sample size was done, while we observed a mean ± standard deviation of shock duration amounting to 3.0 ± 3.8 days in the control group, making the study underpowered to detect significant difference in time to shock resolution. Nonetheless, based on the lower bound of the 95% confidence interval of the difference in median time to shock resolution between groups, a maximal reduction of 0.8 days of shock duration may be anticipated with the use of preload dependence indices, a difference that may not be clinically relevant. Third, one may question that the control group reflects the standard of care, as the surviving sepsis campaign now recommends CVP-guided fluid loading in the early phase of septic shock resuscitation and fluid challenge thereafter [1]. The target CVP level chosen in the present study is another questionable matter. A higher CVP target (that is 12 to 15 mm Hg) has been advocated in patients under mechanical ventilation or with preexisting decreased ventricular compliance [1]. Nevertheless, should this recommendation be applied in the present study, it would have increased the amount of fluids given to the control group, and hence increase the difference with the intervention group, regarding this parameter. Four, PLR was performed from the supine position in the present study, while one study suggested that starting from the semi-recumbent position increased the diagnosis performance of the PLR test [29]. However, a meta-analysis of nine studies did not find any effect of the starting position on the diagnosis accuracy of the PLR test [11]. Five, treatment arm blinding was not possible in the present study, and observer bias was hence uncontrolled, which may have influenced the main judgment criterion. Nevertheless, vasopressor weaning was strictly protocolled, performed by ICU nurses, and none of the investigators was involved in vasopressor tapering. Furthermore, no between-group differences in non-hemodynamic treatment could be documented (see Additional file 3). Six, external validity of the study may be questionable because of the relatively low inclusion to screening ratio (10%). Seven, evaluation of tissue hypoperfusion as an incentive to trigger intravascular volume expansion was not performed, since many clinical or biological variables may be used at the bedside (elevated lactate, low urine output, low ScvO_2_, tachycardia, mottling, metabolic acidosis, increased capillary refill time, low cardiac output, among others) and explicit implementation of these variables into an hemodynamic protocol to avoid co-intervention bias in a non-blinded study raises issues such as interobserver variability (mottling, capillary filling time), or explicit definition of cutoff values (cardiac rate, pH, cardiac output). Nevertheless, the lack of tissue perfusion evaluation before fluid bolus was applied in both study arms and may not interfere with the effect (or lack of effect) on outcome. Eight, the study was erroneously registered after the inclusion of the last patient, ending up in a theoretical selective outcome reporting bias. Finally, we observed a relatively high mortality in the control group. However, mortality of the present study was in the range of recently published randomized controlled studies on severe sepsis (see Additional file 6), while patient severity, comorbidities and rate of immunosuppressed patients were in the upper range, and exclusion criteria of patients with high expected mortality were less stringent in the present study.

Our study has, however, several strengths. First, the design, with protocolled hemodynamic management and associated treatments for septic shock, made intravascular volume expansion strategies the only distinct intervention between both treatments arms, allowing a direct comparison of the two fluid-loading strategies regarding their effect on patient outcome. Second, each hemodynamic strategy was applied for the whole duration of septic shock, from the early phase to the vasopressor withdrawal, contrary to previous studies that tested hemodynamic interventions applied in the first 6 to 8 hours of management [4-7]. Third, we observed a strong adherence to the hemodynamic protocol during the whole study period. Fourth, the study population was characterized with a high prevalence of bacterial documentation and raised arterial lactate level at inclusion,

### Effect of preload dependence-driven intravascular volume expansion on plasma lactate

The beneficial effect on lactate decrease, that is the faster rate of decline observed in the preload dependence group, is in line with previous studies using dynamic preload indices performed in the perioperative setting [12-15]. In contrast to some of the aforementioned studies [12,14,15], but in line with another one [13], the effect on lactate decrease was achieved in the present study with less intravascular fluids administration. However, this effect was identified on *post hoc* time-dependant analysis, while the predefined criterion (number of days with greater than normal plasma lactate) was not statistically significant. Therefore, these results should be addressed with caution and only viewed as exploratory for future studies.

### Effect of preload dependence-driven intravascular volume expansion on fluid administration

An important finding of the present study is that driving intravascular fluid administration with preload dependance indices reduced the daily amount of administered fluids with, at most, no detrimental effect on outcome. The significant decrease in RBC transfusion requirement in the preload dependence arm may be a consequence of the lower amount of fluid administered in this group since hemoglobin levels were not significantly different between groups. Another important finding of this study is that preload dependence-driven fluid resuscitation resulted in a stepwise increase in the amount of administered fluids as patient severity increased (Figure 5), as opposed to CVP-driven resuscitation. This suggests that such strategy may help to tailor fluid administration, by selecting the most severe patients for aggressive fluid resuscitation, and emphasizes the lack of efficacy of CVP-driven resuscitation for that purpose.

### Effects on outcomes

The present study did not find any significant effect of the intervention arm on shock duration, or any end point related to outcome. The interaction between patient severity and fluid administration may explain this finding since half of the intervention arm population was exposed to a more restrictive fluid resuscitation with no expected effect on shock duration and an expected beneficial effect on outcome criteria related to volume overload (the less severe patients), while the opposite was true in the more severe patients (Figure 5), making the study underpowered to detect significant differences.

### Clinical relevance of PPV

While we originally planned a combined use of PPV and PLR test to trigger fluid administration since many clinical scenarios preclude the use of PPV to test fluid responsiveness [30-33], we observed that PPV validity criteria were marginally present in the study patients, mainly because of the strong adherence to low V_T_ ventilation (see Additional file 3), suggesting that PPV may be of little clinical use in the management of septic shock patients.

## Conclusions

To sum up, in patients with septic shock titrating intravascular volume expansion with preload dependence indices did not change time to shock resolution but resulted in less daily fluids intake, including red blood cells, without worsening patient outcome.

## Key messages

- In septic shock patients, titrating intravascular volume expansion with preload dependence indices may have no effect on time to shock resolution.
- This strategy is, however, associated with a decrease in both the daily amount of intravascular fluids and red blood cell transfusion, with no outcome penalty.

## Additional files

Additional file 1:
**Fluid administration.**
Additional file 2:
**Evolution of hemoglobin levels over time.**
Additional file 3:
**Associated treatments.**
Additional file 4:
**Causes of death and limitation of treatment.**
Additional file 5:
**Protocol violations regarding intravascular volume expansion.**
Additional file 6:
**Severe sepsis randomized controlled trials published since 2010.**

## Abbreviations

ACP: acute cor pulmonale
CI: cardiac index
CVP: central venous pressure
ELWI: extravascular lung water index
FiO_2_: inspired oxygen fraction
Ht: hematocrit
ICU: intensive care unit
IV: intravenous
MAP: mean arterial pressure
MV: mechanical ventilation
NS: non-statistically significant
PaO_2_: partial pressure of arterial oxygen
PBW: predicted body weight
PEEP: positive end-expiratory pressure
PLR: passive leg raising
PPV: pulse pressure variation
RBC: red blood cell
SAPS II: simplified acute physiology score II
ScvO_2_: superior vena cava oxygenation saturation
SOFA: sequential organ failure assessment
SR: sinus rhythm
SRM: spontaneous respiratory movements
ΔSV: stroke volume variation after fluid administration
V_T_: tidal volume
