# Supplementary material for: Preload dependence indices to titrate volume expansion during septic shock: a randomized controlled trial
Source: Crit Care. 2015 Jan 8;19(1):5. doi: 10.1186/s13054-014-0734-3 (PMC4310180; doi:10.1186/s13054-014-0734-3)
Supplement: Additional file 1: — Fluid administration. [file 13054_2014_734_MOESM1_ESM.docx]

Additional file 1

**Title**: Fluid administration

**Description of data**: Fluid administration as a function of time from inclusion according to study arm.

|  | Control group | Preload dependence group | p | At risk - Control group | At risk - Preload dependence group |
| --- | --- | --- | --- | --- | --- |
| Intravascular volume expansion H0-H6 (mL) | 500 [0-2500] | 0 [0-500] | 0.02 | 30 | 30 |
| Intravascular volume expansion H6-H12 (mL) | 0 [0-500] | 0 [0-500] | 0.56 | 30 | 30 |
| Intravascular volume expansion H12-H24 (mL) | 300 [0-500] | 300 [0-1000] | 0.82 | 29 | 29 |
| Intravascular volume expansion H24-H48 (mL) | 500 [0-1000] | 100 [0-750] | 0.39 | 24 | 27 |
| Intravascular volume expansion H48-H72 (mL) | 500 [0-1000] | 850 [500-1250] | 0.26 | 18 | 20 |
| Intravascular volume expansion H0-H72 (mL) | 2900 [1625-5775] | 2850 [950-3550] | 0.65 | 18 | 20 |
| Intravascular volume expansion H0-EOS (mL) | 3300 [2350-5500] | 3000 [1000-5900] | 0.53 | 30 | 30 |

EOS = end of study.
