# Supplementary material for: Preload dependence indices to titrate volume expansion during septic shock: a randomized controlled trial
Source: Crit Care. 2015 Jan 8;19(1):5. doi: 10.1186/s13054-014-0734-3 (PMC4310180; doi:10.1186/s13054-014-0734-3)
Supplement: Additional file 2: — Evolution of hemoglobin levels over time. [file 13054_2014_734_MOESM2_ESM.docx]

Additional file 2

**Title**: Evolution of hemoglobin levels over time.

**Description of data**: Evolution of hemoglobin levels over time according to study group.

Symbols are mean parameters value over time (blue = control group, red = preload dependence group). Bars are standard deviation.

NS = non significant.
