# Supplementary material for: Preload dependence indices to titrate volume expansion during septic shock: a randomized controlled trial
Source: Crit Care. 2015 Jan 8;19(1):5. doi: 10.1186/s13054-014-0734-3 (PMC4310180; doi:10.1186/s13054-014-0734-3)
Supplement: Additional file 3: — Associated treatments. [file 13054_2014_734_MOESM3_ESM.docx]

Additional file 3

**Title**: Associated treatments.

**Description of data**: Non-hemodynamic treatments administered to patients according to study arm.

|  | Control  (n=30) | Preload dependence (n=30) | p |
| --- | --- | --- | --- |
| Duration of antibiotics treatment (d) | 8 [5-15] | 12 [5-15] | 0.30 |
| Patients under sedation more than 24 hours | 15 (50%) | 17 (57%) | 0.80 |
| Days under sedation (% of study duration) | 11 [0-68] | 20 [0-58] | 0.88 |
| Median daily V_T_ (ml.kg^-1^ PBW) | 6.0 [6.0-6.1] | 6.1 [6.0-7.0] | 0.07 |
| Median PEEP (cm H_2_O) | 8 [5-10] | 5 [5-8] | 0.25 |
| Patients treated with prone position for ARDS | 5 (17%) | 1 (3%) | 0.20 |
| Patients treated with furosemide | 17 (57%) | 15 (50) | 0.80 |
| Days under furosemide (% of study duration) | 14 [0-48] | 4 [0-50] | 0.77 |
| Furosemide dose (mg per day of treatment) | 60 [40-107] | 60 [43-72] | 1 |
| Patients treated with low dose steroids | 29 (97%) | 29 (97%) | 1 |
| Days under low dose steroids (% of study duration) | 82 [62-100] | 90 [69-100] | 0.58 |
| Low dose steroids dose (mg per day of treatment) | 200 [200-200] | 200 [188-200] | 0.11 |
| Median daily glycaemia (mmol.L^-1^) | 8.0 [7.4-9.5] | 8.1 [7-9.3] | 0.6 |
| Glycaemia > 10 mmol.L^-1^ (% of total number of measurements) | 20 [0-42] | 18 [0-38] | 0.72 |
| Patients treated with rhACP | 0 (0%) | 0 (0%) | 1 |
| Patients treated with therapeutic heparin | 18 (60%) | 14 (47%) | 0.44 |

ARDS = acute respiratory distress syndrome; PBW = predicted body weight; PEEP = positive end-expiratory pressure; rhACP=recombinant human activated C protein; V_T_ = tidal volume.
