# Supplementary material for: Preload dependence indices to titrate volume expansion during septic shock: a randomized controlled trial
Source: Crit Care. 2015 Jan 8;19(1):5. doi: 10.1186/s13054-014-0734-3 (PMC4310180; doi:10.1186/s13054-014-0734-3)
Supplement: Additional file 4: — Causes of death and limitation of treatment. [file 13054_2014_734_MOESM4_ESM.docx]

Additional file 4

**Title**: Causes of death and limitation of treatment.

**Description of data**: Causes of death and limitation of treatment according to study arm.

|  | Control  (n=30) | Preload dependence (n=30) | p |
| --- | --- | --- | --- |
| Causes of death:   - refractory shock - acute respiratory failure - other | 10 (71%)  2 (14%)  2 (14%) | 6 (86%)  0 (0%)  1 (14%) | 0.78 |
| End of life limitation of treatment within 28 days :   - in ICU - after ICU - no limitation | 0 (0%)  3 (21%)  11 (79%) | 0 (0%)  1 (14%)  6 (86%) | 1 |

ICU = intensive care unit.
