# Supplementary material for: Preload dependence indices to titrate volume expansion during septic shock: a randomized controlled trial
Source: Crit Care. 2015 Jan 8;19(1):5. doi: 10.1186/s13054-014-0734-3 (PMC4310180; doi:10.1186/s13054-014-0734-3)
Supplement: Additional file 5: — Protocol violations regarding intravascular volume expansion. [file 13054_2014_734_MOESM5_ESM.docx]

Additional file 5

**Title**: Protocol violations regarding intravascular volume expansion.

**Description of data**: Protocol violations regarding intravascular volume expansion according to study arm.

|  | Control  (n=30) | Preload dependence (n=30) | p |
| --- | --- | --- | --- |
| Number of IVE procedures in excess in violation of the protocol (/day) | 0 [0-0.1] | 0 [0-0.2] | 0.26 |
| Volume administered for IVE in violation of the protocol (mL/day) | 0 [0-53] | 0 [0-95] | 0.26 |
| Total volume administered for IVE in violation of the protocol (mL) | 0 [0-375] | 0 [0-500] | 0.25 |
| Number of IVE procedures not performed in violation of the protocol (/day) | 0 [0-0.0] | 0 [0-0] | 0.59 |
| Total number of protocol violations (/day) | 0 [0-0.2] | 0 [0-0.3] | 0.23 |
| Number of patients with more than 1 protocol violation | 9 (30%) | 13 (43%) | 0.42 |

IVE = intravascular volume expansion.
