# Supplementary material for: Preload dependence indices to titrate volume expansion during septic shock: a randomized controlled trial
Source: Crit Care. 2015 Jan 8;19(1):5. doi: 10.1186/s13054-014-0734-3 (PMC4310180; doi:10.1186/s13054-014-0734-3)
Supplement: Additional file 6: — Severe sepsis randomized controlled trials published since 2010. [file 13054_2014_734_MOESM6_ESM.docx]

Additional file 6

**Title**: Severe sepsis randomized controlled trials published since 2010.

**Description of data**: Main characteristics of severe sepsis randomized controlled trials published since 2010.

| Study | Septic shock | SAPSII | SOFA | APACHE II | Charlson score | Immunodeficiency | Exclusion of patients with high expected mortality | Mortality  Control group | Mortality  intervention group |
| --- | --- | --- | --- | --- | --- | --- | --- | --- | --- |
| Annane [1] | 100% | 59 | 11 | NR | NR | NR | None | HM: 45% | HM: 45% |
| Jones [2] | 82% | 45 | 7 | NR | NR | NR | None | HM: 34% | HM: 25% |
| Schortgen [3] | 100% | NR | 11 | NR | NR | NR | RRT | ICUM: 43% | ICUM: 35% |
| Trof [4] | 100% | NR | 10 | 27 | NR | NR | None | 28dayM: 38% | 28dayM: 42% |
| Perner [5] | 84% | 50 | 7 | NR | NR | NR | RRT | 28dayM: 36% | 28dayM: 39% |
| Ranieri [6] | 100% | NR | NR | 25 | NR | NR | Severe coagulation failure | 28dayM: 24% | 28dayM: 26% |
| Annane [7] | 100% | 56 | 11 | NR | NR | NR | Chronic liver disease  Severe coagulation failure | 28dayM: 35% | 28dayM: 37% |
| Morelli [8] | 100% | 55 | NR | NR | NR | NR | Severe cardiac dysfunction | 28dayM: 81% | 28dayM: 49% |
| ProCESS [9] | 100% | NR | NR | 21 | 2.6 | NR | CD4 < 50 mm^-3^ | NR | NR |
| Caironi [10] | 63% | 48 | 8 | NR | NR | 13% | Congestive heart failure | 28dayM: 32% | 28dayM: 32% |
| Asfar [11] | 100% | 57 | 11 | NR | NR | NR | None | 28dayM: 34% | 28dayM: 37% |
| Present study | 100% | 56 | 11 | 30 | 3 | 33% | None | 28dayM: 47% | 28dayM: 23% |

28dayM = 28 day mortality; HM = hospital mortality; ICUM = intensive care unit mortality; NR = not reported; RRT = renal replacement therapy.

# REFERENCES

1. Annane D, Cariou A, Maxime V, Azoulay E, D’Honneur G, Timsit JF, Cohen Y, Wolf M, Fartoukh M, Adrie C, Santre C, Bollaert PE, Mathonet A, Amathieu R, Tabah A, Clec’h C, Mayaux J, Lejeune J, Chevret S: **Corticosteroid treatment and intensive insulin therapy for septic shock in adults: a randomized controlled trial**. *JAMA* 2010, **303**:341–8.

2. Jones AE, Shapiro NI, Trzeciak S, Arnold RC, Claremont HA, Kline JA: **Lactate clearance vs central venous oxygen saturation as goals of early sepsis therapy: a randomized clinical trial**. *JAMA* 2010, **303**:739–46.

3. Schortgen F, Clabault K, Katsahian S, Devaquet J, Mercat A, Deye N, Dellamonica J, Bouadma L, Cook F, Beji O, Brun-Buisson C, Lemaire F, Brochard L: **Fever Control Using External Cooling in Septic Shock: a Randomized Controlled Trial**. *Am J Respir Crit Care Med* 2012, **185**:1088–95.

4. Trof RJ, Beishuizen A, Cornet AD, de Wit RJ, Girbes ARJ, Groeneveld ABJ: **Volume-limited versus pressure-limited hemodynamic management in septic and nonseptic shock**. *Crit Care Med* 2012, **40**:1177–1185.

5. Perner A, Haase N, Guttormsen AB, Tenhunen J, Klemenzson G, Åneman A, Madsen KR, Møller MH, Elkjær JM, Poulsen LM, Bendtsen A, Winding R, Steensen M, Berezowicz P, Søe-Jensen P, Bestle M, Strand K, Wiis J, White JO, Thornberg KJ, Quist L, Nielsen J, Andersen LH, Holst LB, Thormar K, Kjældgaard A-L, Fabritius ML, Mondrup F, Pott FC, Møller TP, et al.: **Hydroxyethyl Starch 130/0.42 versus Ringer’s Acetate in Severe Sepsis**. *N Engl J Med* 2012, **367**:124–134.

6. Ranieri VM, Thompson BT, Barie PS, Dhainaut JF, Douglas IS, Finfer S, Gardlund B, Marshall JC, Rhodes A, Artigas A, Payen D, Tenhunen J, Al-Khalidi HR, Thompson V, Janes J, Macias WL, Vangerow B, Williams MD: **Drotrecogin Alfa (Activated) in Adults with Septic Shock**. *N Engl J Med* 2012, **366**:2055–64.

7. Annane D, Timsit J-F, Megarbane B, Martin C, Misset B, Mourvillier B, Siami S, Chagnon J-L, Constantin J-M, Petitpas F, Souweine B, Amathieu R, Forceville X, Charpentier C, Tesnière A, Chastre J, Bohe J, Colin G, Cariou A, Renault A, Brun-Buisson C, Bellissant E, APROCCHSS Trial Investigators: **Recombinant human activated protein C for adults with septic shock: a randomized controlled trial**. *Am J Respir Crit Care Med* 2013, **187**:1091–1097.

8. Morelli A, Ertmer C, Westphal M, Rehberg S, Kampmeier T, Ligges S, Orecchioni A, D’Egidio A, D’Ippoliti F, Raffone C, Venditti M, Guarracino F, Girardis M, Tritapepe L, Pietropaoli P, Mebazaa A, Singer M: **Effect of heart rate control with esmolol on hemodynamic and clinical outcomes in patients with septic shock: a randomized clinical trial**. *JAMA J Am Med Assoc* 2013, **310**:1683–1691.

9. The ProCESS Investigators: **A Randomized Trial of Protocol-Based Care for Early Septic Shock**. *N Engl J Med* 2014.

10. Caironi P, Tognoni G, Masson S, Fumagalli R, Pesenti A, Romero M, Fanizza C, Caspani L, Faenza S, Grasselli G, Iapichino G, Antonelli M, Parrini V, Fiore G, Latini R, Gattinoni L, the ALBIOS Study Investigators: **Albumin Replacement in Patients with Severe Sepsis or Septic Shock**. *N Engl J Med* 2014, **370**:1412–21.

11. Asfar P, Meziani F, Hamel J-F, Grelon F, Megarbane B, Anguel N, Mira J-P, Dequin P-F, Gergaud S, Weiss N, Legay F, Le Tulzo Y, Conrad M, Robert R, Gonzalez F, Guitton C, Tamion F, Tonnelier J-M, Guezennec P, Linden TVD, Vieillard-Baron A, Mariotte E, Pradel G, Lesieur O, Ricard J-D, Hervé F, Cheyron DD, Guerin C, Mercat A, Teboul J-L, et al.: **High versus Low Blood-Pressure Target in Patients with Septic Shock**. *N Engl J Med* 2014.
